# Patient and physician factors influence decision-making in hypercholesterolemia: a questionnaire-based survey

**DOI:** 10.1186/s12944-015-0037-y

**Published:** 2015-05-19

**Authors:** Michel Krempf, Ross J Simpson, Dena Rosen Ramey, Philippe Brudi, Hilde Giezek, Joanne E Tomassini, Raymond Lee, Michel Farnier

**Affiliations:** Endocrinology and Nutrition, Hôpital Laënnec, Nantes, 44035 France; Center for Heart and Vascular Care, University of North Carolina, Chapel Hill, NC USA; Merck & Co, Inc., Kenilworth, NJ USA; MSD Inc., Brussels, Belgium; Point Medical, 21000 Dijon, France

**Keywords:** Decision-making, Hypercholesterolemia, LDL-C, Ezetimibe, Statins

## Abstract

**Background:**

Goal attainment of guideline-recommended low-density lipoprotein cholesterol (LDL-C) is suboptimal. Little is known about how patient factors influence physicians’ treatment decision-making in hypercholesterolemia. We examined physicians’ treatment recommendations in high-risk patients whose LDL-C remained uncontrolled despite statin monotherapy.

**Methods:**

Physicians completed a questionnaire prior to randomization into period I of a two-period randomized controlled trial evaluating LDL-C goal attainment in patients whose LDL-C remained ≥100 mg/dL after 5 weeks’ treatment with atorvastatin 10 mg/day (NCT01154036). Physicians’ treatment recommendations were surveyed for two hypothetical and one real scenario: (1) LDL-C presumed near goal (between 100–105 mg/dL), (2) LDL-C presumed far from goal (~120 mg/dL), and (3) observed baseline LDL-C of enrolled patients. Prognostic factors considered during decision-making were identified by regression analysis. Observed lipid outcomes at the end of period I (following 6 weeks’ treatment with ezetimibe 10 mg plus atorvastatin 10 mg, atorvastatin 20 mg, or rosuvastatin 10 mg) were compared with estimated LDL-C outcomes for physicians’ treatment recommendations after 6 weeks (based on individual patients’ pre-randomization LDL-C and expected incremental change).

**Results:**

Questionnaires were completed for 1,534 patients. No change in therapy, or double atorvastatin dose, were frequently recommended, even when LDL-C was far from goal (6.5% and 52.2% of patients, respectively). Double atorvastatin dose was commonly recommended in all scenarios (43–52% of patients). More intensive LDL-C-lowering regimens were recommended infrequently e.g. double atorvastatin dose *and* add ezetimibe only <12% in all scenarios. Overall, cardiovascular risk factors and desire to achieve a more aggressive LDL-C goal were prominent factors in decision-making for treatment. Comparison of observed and estimated LDL-C levels showed that physicians tended to overestimate the effectiveness of their recommendations.

**Conclusions:**

This study provides insight into physicians’ perspectives on clinical management of hypercholesterolemia and highlights a gap in knowledge translation from guidelines to clinical practice. The need for lower LDL-C and cardiovascular risk were key drivers in clinical decision-making, but physicians’ treatment choices were more conservative than guideline recommendations, potentially resulting in poorer LDL-C reduction. When compared with actual outcomes, projected LDL-C control was better if physicians used more comprehensive strategies rather than simply doubling the statin dose.

**Trial registration:**

Clinicaltrials.gov: NCT01154036

**Electronic supplementary material:**

The online version of this article (doi:10.1186/s12944-015-0037-y) contains supplementary material, which is available to authorized users.

## Background

Reducing low-density lipoprotein cholesterol (LDL-C) levels with statin therapy is associated with clear benefit in reducing cardiovascular risk [1]. For years, both European and US dyslipidemia guidelines advocated a treat-to-target approach for LDL-C reduction to <100 mg/dL [2,3]. Since the development of this study, American Heart Association/American College of Cardiology guidelines have advocated a shift away from LDL-C treatment targets *per se* to focus on identifying patients most likely to benefit from high-intensity (LDL-C reduction ≥50%) or moderate-intensity (LDL-C reduction 30 - <50%) statin therapy [4]. International guidelines, however, continue to advocate a treat-to-target approach, albeit with a more stringent LDL-C target for very high cardiovascular risk patients of <70 mg/dL or a 50% reduction from baseline, compared with an LDL-C target of <100 mg/dL for high cardiovascular risk patients [5,6].

Many patients at high cardiovascular risk receiving statin monotherapy experience sub-optimal LDL-C-lowering with persistent residual risk [7-9]. Retrospective analysis of medical records for >27,400 US patients with very high cardiovascular risk (e.g., coronary heart disease or atherosclerotic vascular disease) with prescriptions for atorvastatin monotherapy showed that >65% of patients had LDL-C levels >70 mg/dL and, of these, 30–40% had LDL-C ≥20 mg/dL in excess of this level regardless of dose [7]. Where LDL-C-lowering remains sub-optimal on statin monotherapy, guidelines generally recommend using the maximum tolerated statin dose in high-risk individuals [4,5] or introducing combination therapy with another lipid-lowering agent [5,10-12].

In clinical practice, physicians may/should use evidence-based guidelines to implement an individualized sequential treatment approach to lipid management, particularly for relatively challenging high-risk patients who require more intensive LDL-C reduction. Nevertheless, failure to achieve therapeutic LDL-C targets persists for various reasons, including non-adherence, intolerance and cost factors [13-16]. For high-risk patients, including those who may be poor responders and/or intolerant to treatment with higher statin doses, baseline levels can greatly exceed target values making it difficult to achieve LDL-C goals. In addition, achieving target LDL-C may require several steps, such as dose uptitration, or combination therapy; thus, compliance and cost factors can be an issue. Physicians’ knowledge, attitudes, and beliefs also play a major role in the translation of guideline-based evidence to the treatment choices they make in clinical practice [17-19]. Specifically, organizational structures, time constraints, perceived lack of usefulness of guidelines, and knowledge gaps commonly preclude wider implementation of best-practice recommendations [20]. Furthermore, with physician-patient partnerships increasingly promoted in healthcare decision-making, physicians (particularly in general practice) often adopt a pragmatic and flexible approach to guideline implementation in order to preserve relationships with their patients [12,21,22].

To date, there has been little study of patient characteristics that influence physicians’ treatment decisions. We therefore conducted a questionnaire-based survey of physicians participating in a randomized controlled trial (RCT) to better understand physician attitudes/beliefs and patient characteristics that influence clinical decision-making for specific treatments in hypercholesterolemia. The primary results of the RCT, which assessed LDL-C goal attainment rates in high-risk patients with hypercholesterolemia and elevated LDL-C on atorvastatin monotherapy when changed to more potent LDL-C-lowering therapies (i.e., uptitration, ezetimibe add-on, or switch to a more potent statin), have been published previously (NCT01154036) [23].

Here we report the findings of the questionnaire-based survey, which assessed physicians’ treatment recommendations and prognostic factors involved in decision-making for two hypothetical and one real scenario: (1) LDL-C presumed near goal (between 100–105 mg/dL), (2) LDL-C presumed far from goal (~120 mg/dL), and (3) observed baseline LDL-C. The survey was conducted just prior to randomization of each patient into the RCT, thus providing a unique opportunity to compare estimated outcomes based on treatment choices made in a real-life clinical setting with those obtained under strict protocol guidance during an actual RCT.

## Results

### Physician and patient characteristics

Physicians at 296 sites in 29 countries randomized 1,547 patients to receive treatment with ezetimibe 10 mg plus atorvastatin 10 mg, atorvastatin 20 mg, or rosuvastatin 10 mg daily for 6 weeks during period I of a phase III, two-period, multicenter, double-blind RCT [23]; 1,460 of these patients completed period I.

Physicians completed the pragmatic use questionnaire for a total of 1,534 male and female patients at high cardiovascular risk; the majority (67.2%) were aged <65 years and approximately 50% had prior history of cardiovascular disease (CVD) (Table 1). In total, 57.7% of questionnaires were completed by specialist physicians and 42.3% by primary care physicians (Table 2).

**Table 1:** **Patient characteristics (visit 3: one week prior to pre-randomization)**

	**n = 1,534 patients**
Age, n (%)	
<65 years	1031 (67.2)
≥65 years	503 (32.8)
Male, n (%)	728 (47.5)
Prior history of CVD, n (%)	774 (50.5)
LDL-C (mg/dL), mean (SD)	121 (18)
EZE 10 mg + ATV 10 mg	121 (18)
ATV 20 mg	120 (17)
RSV 10 mg	121 (18)
LDL-C (mg/dL), n (%)	
<85	11 (0.7)
85–100	133 (8.7)
100–130	956 (62.3)
130–160	411 (26.8)
≥160	23 (1.5)

ATV, atorvastatin; EZE, ezetimibe; CVD, cardiovascular disease; LDL-C, low-density lipoprotein cholesterol; RSV, rosuvastatin.

**Table 2:** **Physician characteristics**

	**n = 1,534 patients**
Type of physician, n (%)	
Primary care physician	649 (42.3)
Specialist	885 (57.7)
Years of practicing medicine	
<5	152 (9.9)
5–9	201 (13.1)
10–19	367 (23.9)
≥20	814 (53.1)

### Randomized controlled trial

The observed mean (standard deviation) baseline LDL-C (mg/dL) of patients randomized into period I of the RCT was: 121 (18) for ezetimibe 10 mg plus atorvastatin 10 mg, 120 (17) for atorvastatin 20 mg, and 121 (18) for rosuvastatin 10 mg. After 6 weeks’ double-blind treatment, at the end of period I, the percent change from baseline in LDL-C was −20%, −7%, and −11%, for the three respective treatment groups.

### Recommended treatment choice

Among the treatment choices, the four most common recommendations for all three LDL-C scenarios surveyed were: (a) no change in therapy, (b) double the atorvastatin dose, (c) add ezetimibe, and (d) double the atorvastatin dose *and* add ezetimibe, albeit with variability across the three scenarios (Table 3). Notable data relating to the impact of a fifth recommendation – switch to rosuvastatin – is also provided in Table 3. Higher mean baseline LDL-C values tended to be associated with recommendations for more intensive therapy. No change in therapy, clearly the most conservative approach, was associated with the lowest mean LDL-C value. Doubling the atorvastatin dose *and* adding ezetimibe was associated with the highest mean LDL-C level. Mean baseline LDL-C levels were similar in patients recommended to switch to rosuvastatin or add ezetimibe.

**Table 3:** **Most common treatment recommendations by LDL-C scenario**

**Recommendation**	**Scenario (% patients)**			**LDL-C observed BL RCT level (mg/dL)**
	**1**	**2**	**3**	
	**LDL-C presumed near goal**	**LDL-C presumed far from goal**	**LDL-C level known at pre-randomization**	
	**100–105 mg/dL**	**~ 120 mg/dL**		
No change in therapy	42.3	6.5	17.5	111.4
Double ATV dose	42.8	52.2	46.5	119.2
Add EZE	8.7	15.6	15.0	123.7
Double ATV dose *and* add EZE	2.0	11.6	9.3	129.3
Switch to RSV	1.0	4.3	2.6	124.8

Values shown are mean values.

ATV, atorvastatin; BL, baseline; EZE, ezetimibe; LDL-C, low-density lipoprotein cholesterol; RCT, randomized clinical trial; RSV, rosuvastatin.

Doubling the atorvastatin dose was the most common treatment recommendation across all scenarios, recommended for 42.8–52.2% of patients, although physicians recommended no change in therapy in a similar proportion of patients when LDL-C was presumed close to goal (42.3%). Interestingly, no change in therapy was recommended by physicians in approximately 18% of patients, even when they were aware of the patients’ observed baseline LDL-C level. Physicians recommended more intensive or modified LDL-C-lowering regimens when LDL-C was presumed far from versus near to goal. Interestingly, no change in therapy was still recommended in 6.5% of patients when LDL-C was presumed far from goal. In general, treatment recommendations by physicians were conservative: the combined treatment by adding ezetimibe or doubling the atorvastatin dose and adding ezetimibe were recommended infrequently (in <11.6% – 15.6% of patients) in all three scenarios, while switch to rosuvastatin was recommended for fewer than 4.3% of patients when LDL-C was presumed far from goal.

### Factors affecting physicians’ treatment recommendations

**Figure 1:**
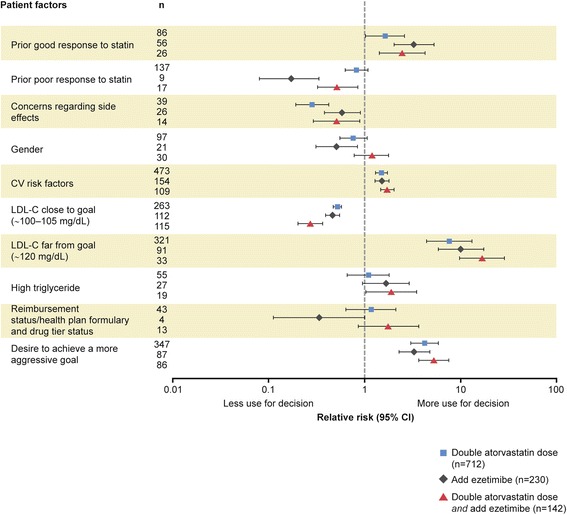
Association of patient factors with physicians’ treatment recommendations relative to observed baseline LDL-C from RCT. Prognostic factors selected by forward stepwise regression model of physician treatment choice, relative to no change in therapy (n = 268), RR (95% CI). RR >1: prognostic factor more likely to be selected by the physician in their treatment choice compared to a choice of no change in therapy; RR <1: prognostic factor less likely to be selected versus no change in therapy. The questionnaire allowed physicians to choose from many treatment options, with some only selected with very low frequency. To provide optimal focus on investigator behavior, the patient factors shown relate only to the four most common treatment choices selected. CI, confidence interval; CV, cardiovascular; LDL-C, low-density lipoprotein cholesterol; RR, relative risk.

**Figure 2:**
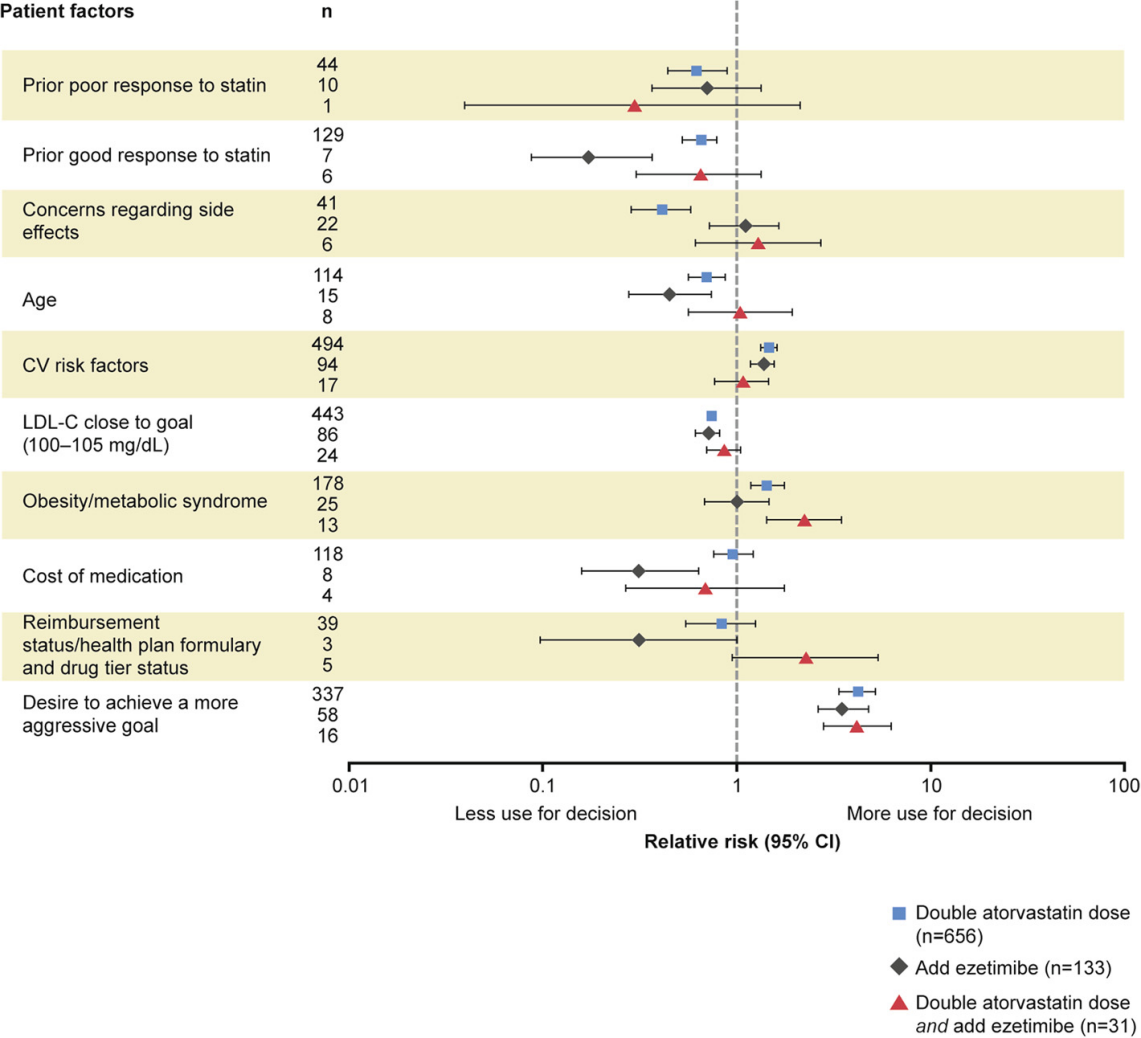
Association of patient factors with physicians’ treatment recommendations relative to LDL-C presumed near to goal (100–105 mg/dL). Prognostic factors selected by forward stepwise regression model of physician treatment choice, relative to no change in therapy (n = 648), RR (95% CI). RR >1: prognostic factor more likely to be selected by the physician in their treatment choice compared to a choice of no change in therapy; RR <1: prognostic factor less likely to be selected versus no change in therapy. The questionnaire allowed physicians to choose from many treatment options, with some only selected with very low frequency. To provide optimal focus on investigator behavior, the patient factors shown relate only to the four most common treatment choices selected. CI, confidence interval; CV, cardiovascular; LDL-C, low-density lipoprotein cholesterol; RR, relative risk.

**Figure 3:**
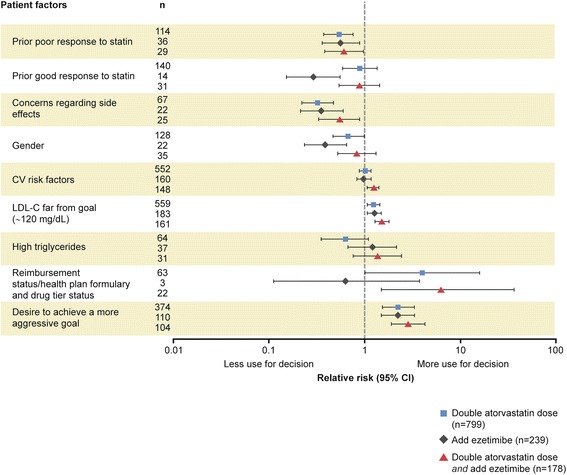
Association of patient factors with physicians’ treatment recommendations relative to LDL-C presumed far from goal (~120 mg/dL). Prognostic factors selected by forward stepwise regression model of physician treatment choice, relative to no change in therapy (n = 100), RR (95% CI). RR >1: prognostic factor more likely to be selected by the physician in their treatment choice compared to a choice of no change in therapy; RR <1: prognostic factor less likely to be selected versus no change in therapy. The questionnaire allowed physicians to choose from many treatment options, with some only selected with very low frequency. To provide optimal focus on investigator behavior, the patient factors shown relate only to the four most common treatment choices selected. CI, confidence interval; CV, cardiovascular; LDL-C, low-density lipoprotein cholesterol; RR, relative risk.

Physician characteristics were relatively unimportant to treatment recommendations in any of the given scenarios (data not shown). When compared with no change in therapy, cardiovascular risk factors and the desire to achieve an even lower LDL-C goal were the most prominent prognostic factors considered to impact physician decision-making, irrespective of active treatment recommendation or LDL-C scenario (Figures 1, 2 and 3; Additional file 1). Cardiovascular risk factors and desire to achieve a more aggressive LDL-C goal were also the factors most likely to be considered in decision-making by physicians when recommending more intensive treatment i.e., double atorvastatin dose, add ezetimibe, or double atorvastatin dose *and* add ezetimibe (Figures 1, 2 and 3; Additional file 1). Prior response to statin (whether poor or good), reimbursement status, LDL-C far from goal, and LDL-C close to goal were also patient factors that contributed strongly, but variably, to decision-making. Other prognostic factors included obesity, cost of medication, age, high triglyceride, and gender (Figures 1, 2 and 3; Additional file 1). Concerns regarding side effects were considered more often by physicians when recommending no change in therapy than other treatments, in particular for the scenarios of LDL-C presumed far from goal and baseline LDL-C known.

### Estimated lipid outcomes (with physician-recommended treatments) versus randomized controlled trial lipid outcomes

**Figure 4:**
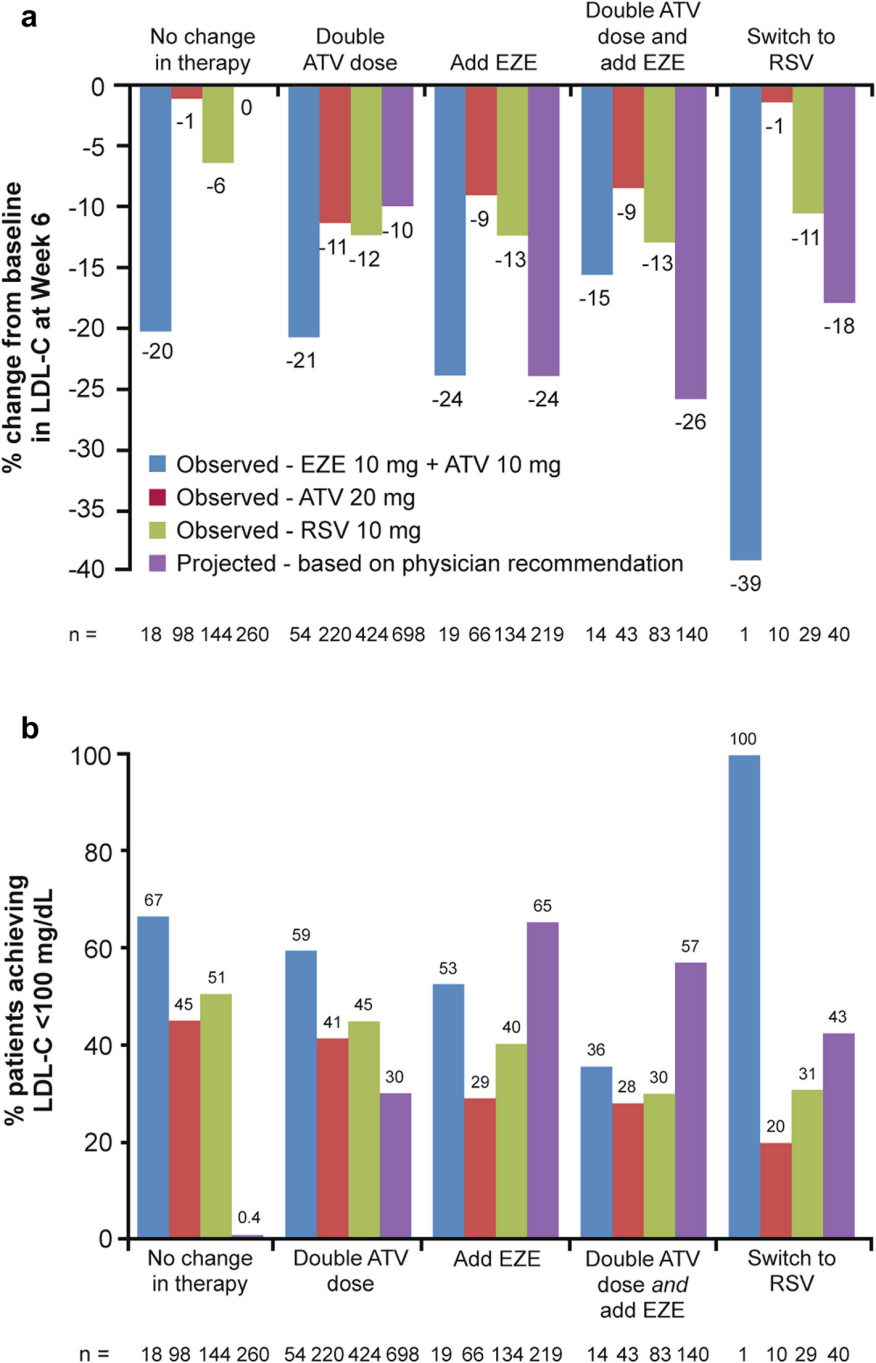
LDL-C outcomes: estimated change from baseline by physician recommendation versus actual observed RCT LDL-C. **(a)** Percent change from baseline in LDL-C at Week 6 **(b)** Percent patients achieving LDL-C <100 mg/dL. Observed (RCT) outcomes at end of period I for randomized treatment. Estimated outcomes of physician-recommended treatments were based on expected reductions for treatment-naïve patients in product labels and literature. Estimated outcomes were calculated based on % incremental benefit expected if the recommended treatment were applied to the observed LDL-C value at the end of the run-in phase for each patient treated with atorvastatin 10 mg; individual estimates were averaged across all patients where a particular treatment was recommended. The estimated proportion of patients achieving a treatment goal (<100 or <70 mg/dL) was derived by applying each patient’s estimated LDL-C change associated with the recommended treatment. ATV, atorvastatin; EZE, ezetimibe; LDL-C, low-density lipoprotein cholesterol; RCT, randomized controlled trial; RSV, rosuvastatin.

As expected, patients receiving a relatively intensive treatment regimen in the RCT showed the largest LDL-C reductions. After 6 weeks’ double-blind treatment (i.e. at the end of period I), the observed reduction from baseline in LDL-C in patients randomized to ezetimibe 10 mg + atorvastatin 10 mg was −21%. This compared with an estimated reduction (based on patients’ pre-randomization LDL-C and expected incremental change in LDL-C 6 weeks after initiation of recommended treatment choice) of only −10% when physicians recommended doubling of atorvastatin dose to 20 mg (Figure 4a). Similarly, the number of patients receiving ezetimibe 10 mg + atorvastatin 10 mg in the RCT who achieved LDL-C <100 mg/dL was greater than the estimated number of patients achieving the same goal when physicians recommended doubling the atorvastatin dose to 20 mg (59% vs 30%, respectively) (Figure 4b and Additional file 2).

Notably, the observed reduction in LDL-C for ezetimibe 10 mg + atorvastatin 10 mg (i.e., add ezetimibe) in the RCT was the same as that estimated when physicians recommended this treatment (both −24%). In patients where double atorvastatin dose was recommended, 30% were estimated to achieve goal, which was lower than the proportion that were observed to achieve goal during the RCT (41–59%) (Figure 4). In contrast, when add ezetimibe was recommended by physicians, the number of patients estimated to reach LDL-C goal (65%) was greater than observed during the RCT for patients receiving ezetimibe 10 mg + atorvastatin 10 mg (53%), atorvastatin 20 mg (29%), or rosuvastatin 10 mg (40%). In patients recommended to receive double atorvastatin dose and add ezetimibe, the estimated reduction in LDL-C (26%) was also higher than observed for the studied treatment regimens during the RCT (−9 to −15%). Similarly, the proportion of these patients estimated to achieve LDL-C <100 mg/dL (57%) was higher than observed in the RCT (28–36%).

**Figure 5:**
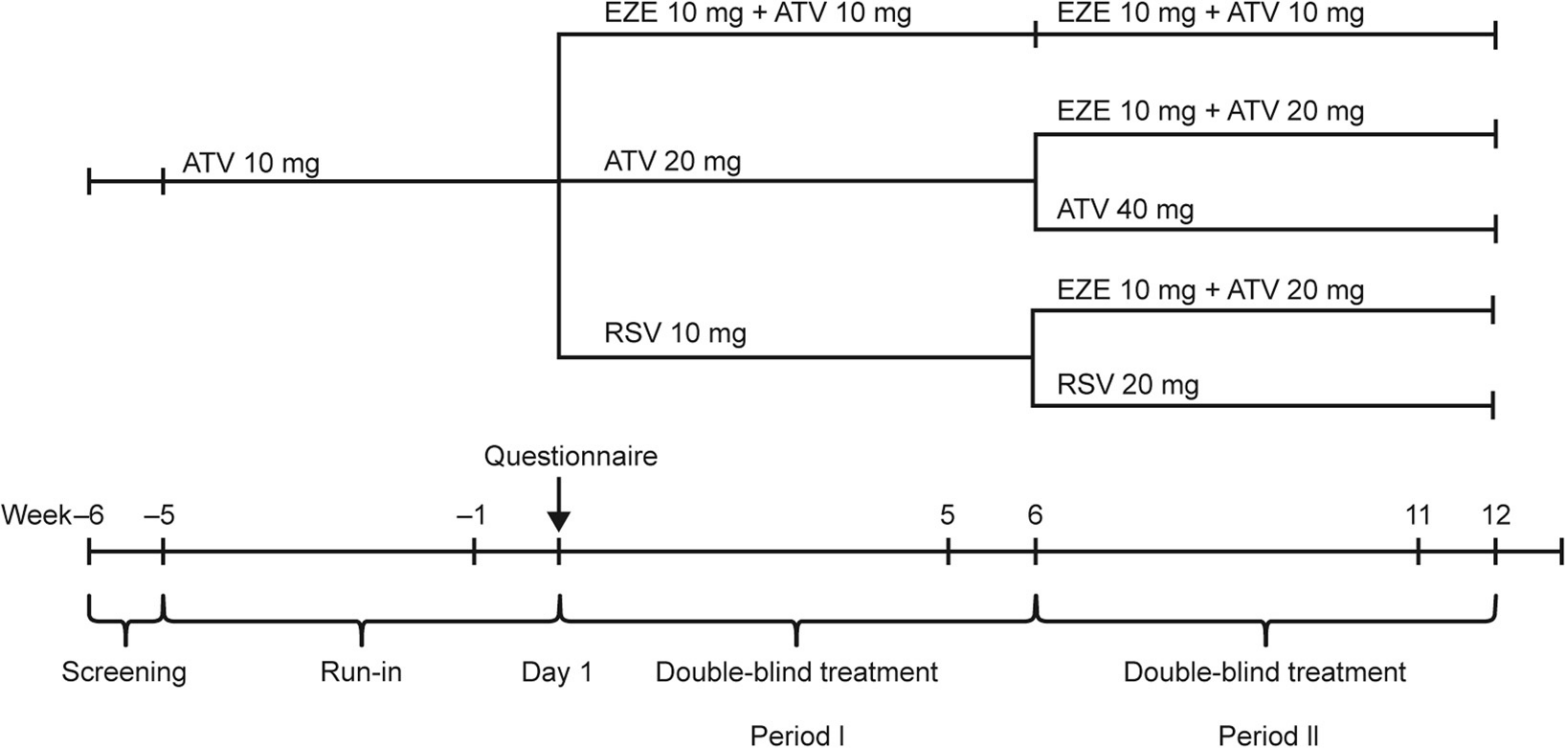
Original primary study design. In the original RCT, LDL-C was determined at Week 5 (Period I) to determine eligibility for Period II of the RCT. At Week 6, patients whose LDL-C levels remained elevated (≥100 and ≤160 mg/dL) had their treatment changed to a more intensive regimen during Period II. Period II baseline LDL-C was the average of Week 5 and 6 data. Lipids were also assessed at Week 11 and 12. Final period II values were the average of Week 11 and 12 data. For this analysis, only period I data were used. For further details of the study design, see Bays et al. 2013 [23]. ATV, atorvastatin; EZE, ezetimibe; RSV, rosuvastatin.

In the original RCT, patients whose LDL-C remained elevated (≥100 mg/dL) at the end of period I changed to a more intensive treatment during period II (Figure 5) [23]. The addition of ezetimibe 10 mg to atorvastatin 20 mg for 6 weeks during period II produced significantly greater reductions from baseline in LDL-C than doubling the atorvastatin dose to 40 mg (−16% vs −6%; p < 0.001) and a significantly greater proportion of patients attained LDL-C <100 mg/dL (56% vs 34%; p < 0.001) [23]. Similarly, switching from rosuvastatin 10 mg to ezetimibe 10 mg + atorvastatin 20 mg produced significantly greater reductions in LDL-C from baseline than doubling the rosuvastatin dose to 20 mg (−15% vs −6%; p < 0.001) and significantly more patients attained LDL-C <100 mg/dL (54% vs 36%; p < 0.001).

## Discussion

This survey provided a unique opportunity to investigate real-world factors that most influence physicians’ choice of lipid-lowering therapy. Physicians’ treatment recommendations were solicited for individual patients who had received atorvastatin 10 mg during a 5-week pre-treatment period and were thus eligible for inclusion in a double-blind RCT designed to assess LDL-C goal attainment rates with different treatment strategies [23]. As such, the survey provides real-world insight into the key drivers used by physicians in the decision-making process for lipid management in patients with hypercholesterolemia.

A number of key determinants in the physician decision-making process, primarily cardiovascular risk factors and the desire to achieve a more aggressive LDL-C goal, were identified in this survey. Notably, patients randomized to a relatively intensive treatment regimen tended to have better LDL-C-lowering outcomes but in practice physicians tended to be conservative, recommending relatively few patients to receive such treatments and typically only when LDL-C was far from goal.

Observational data suggest that, in clinical practice, more potent LDL-C-lowering efficacy interventions involving the combination of a statin with ezetimibe and/or statin uptitration are infrequently prescribed to patients at high cardiovascular risk whose LDL-C remains elevated on low- to moderate-potency statin monotherapy [24,25]. In this questionnaire-based study, physicians were similarly conservative when choosing a regimen for patients who had not responded adequately to initial treatment with atorvastatin 10 mg, especially if such patients were close to, but not at, the recommended goal.

Physicians recommended doubling the atorvastatin dose in approximately 42–50% of patients, regardless of whether or not the observed baseline LDL-C was known or presumed to be near or far from goal. Guidelines generally recommend that the maximum tolerated statin dose should be used in individuals at high cardiovascular risk [3,4], although it is recognized that patients may not achieve adequate LDL-C-lowering on statin therapy alone, with LDL-C being typically reduced only by an additional 4–7% when the statin dose is doubled [26,27]. Adverse events, including myopathy, rhabdomyolysis, and adverse effects on liver enzymes, can also be increased at relatively high statin doses [28]. Consequently, combination therapy with standard statin doses and other lipid-lowering agents is necessary for individuals whose therapeutic response to statins is sub-optimal and if the risk-benefit balance is favorable as recommended in guidelines [4,5,10-12].

We found that when patients’ observed baseline LDL-C was known to physicians, or when LDL-C was presumed to be far from goal, physicians recommended add-on therapy with ezetimibe in only 15% of patients. Addition of ezetimibe 10 mg to ongoing statin therapy has been shown to reduce LDL-C by an additional 14–22% compared with doubling the statin dose [29-34]. In the RCT in which this questionnaire-based study was a component [23], daily treatment for 6 weeks with ezetimibe 10 mg + atorvastatin 10 mg was associated with reduction in LDL-C of −20% from baseline for patients not controlled with atorvastatin 10 mg. This is comparable with that expected, and significantly better than either atorvastatin uptitration or a switch to rosuvastatin in terms of LDL-C change [23]. It is likely that the conservative approach to use of combination therapy seen in clinical practice has, until recently, reflected a lack of confidence amongst physicians in the clinical benefit of LDL-C-lowering by combination therapy due to lack of evidence showing benefits on CVD-related events in outcome trials – both fibrates and niacin, for example, have reported negative trial results [35-37]. While the SHARP (Study of Heart And Renal Protection) trial demonstrated that combination therapy with statin (simvastatin 20 mg) plus ezetimibe 10 mg was associated with a significant decrease in major atherosclerotic events, non-hemorrhagic stroke, and arterial revascularization procedures compared with placebo in patients with severe chronic kidney disease, a statin monotherapy arm was not included in the SHARP study design [38]. The recently completed IMPROVE-IT trial assessed the cardiovascular benefit of LDL-C-lowering with ezetimibe 10 mg added to simvastatin (mainly 40 mg) compared with simvastatin monotherapy in patients presenting with acute coronary syndromes. The study investigators reported that the trial met its primary and secondary composite cardiovascular efficacy endpoints [39-41].

Clinical inertia or conservatism is an increasingly recognized factor in poor management of chronic conditions, such as dyslipidemia and type 2 diabetes [19,42]. In certain complex clinical situations, rather than clinical inertia, it is possible that appropriate inaction may reflect the need for individualized treatment (i.e. patients with multiple comorbidities receiving multiple medications) [19]. There is a need to examine the complex interplay between provider-, patient-, and system-level barriers that contribute to clinical inertia [42].

Our survey enabled identification of prognostic factors considered by physicians during decision-making in hypercholesterolemia. We found that physician characteristics had no evident impact on physician treatment recommendations. No change in therapy was a common recommendation and was even made for 6.5% of patients in the hypothetical scenario of LDL-C being far from goal. Relative to no change in therapy, cardiovascular risk factors and the desire to achieve a more aggressive LDL-C goal were the prognostic factors that appeared to consistently contribute to the physician decision-making process across all scenarios. Specifically, the desire to achieve a more aggressive LDL-C goal was the strongest factor with a consistently high relative risk (RR) across all scenarios and treatment options. Concerns regarding side effects also had an impact on decision-making; this factor was selected more often by the physician when their recommendation was no change in therapy, compared to the other treatment recommendations.

Prior response to a statin was a key factor across all scenarios influencing implementation of combination therapy with statin and ezetimibe. Consideration of reimbursement status generally impacted across all scenarios and treatment recommendations, being a key determinant towards use of combination therapy. An overall apparently counter-intuitive desire to achieve a more aggressive LDL-C goal and apparent reluctance by physicians to implement combination therapy may mirror lack of evidence supporting the use of more potent LDL-C-lowering (combination) therapy in specific patient groups. This observation is, in fact, consistent with findings in other studies showing less frequent use of switching to more potent statin or combination therapy in real-world clinical practice compared with use of moderate-potency lipid-lowering therapy; this is very likely attributed to patient non-compliance/intolerance, health-provider non-adherence to current guidelines, and/or cost factors [13,43]. The situation may be countered by recent reports from IMPROVE-IT [39-41]. Other approaches may include benchmarking, which has been shown to provide a potential means to drive quality of care and improve target attainment of modifiable risk factors in type 2 diabetes [44].

A key strength of this study is that questionnaires were provided prior to treatment results. The data reflects unbiased physician perspectives for clinical management of dyslipidemia. Physicians were more conservative in their treatment choice than guidelines generally recommend. Physicians recommended more aggressive treatment regimens if LDL-C was far from the goal, and estimated lipid outcomes with these recommendations approached or surpassed those attained with the more aggressive randomized treatments. Indeed, when compared with observed lipid outcomes, estimated LDL-C control was better if physicians were to have used more comprehensive strategies rather than simply doubling the statin dose. This is further consistent with the observed LDL-C outcomes from period II of the RCT where more intensive lipid-lowering regimens, such as add-on therapy with ezetimibe, produced incremental improvements in LDL-C-lowering and LDL-C goal attainment in patients who had persistently elevated LDL-C levels following 6 weeks’ of therapy with more conservative strategies, such as atorvastatin 20 mg or rosuvastatin 10 mg [23].

Several factors may be considered limitations. Differing health care systems may influence treatment practices. Through willingness to participate in the trial, it might be expected the primary care physicians and specialists in our study sample would have a greater interest in lipid management than those in broader clinical practice, and may be more familiar with best-practice guidelines. However, this did not seem to have a major influence on the findings as the original RCT design resulted in uptitration and/or switching to a more potent statin in more than 90% of patients [23], whereas 18% of physicians recommended no change in therapy, less than half recommended uptitration and relatively few (<3%) endorsed switching to a more potent statin, even when the baseline LDL-C was known. We believe that in clinical practice, where various real-life patient factors (e.g., age, social environment, comorbid conditions, likelihood of adherence) affect physicians’ prescribing patterns, most physicians would be even more conservative in their treatment approach.

## Conclusion

This study provides insight into physicians’ perspectives on the clinical management of hypercholesterolemia and highlights a gap in guideline implementation. Specifically, physicians adopt a generally conservative approach towards treating patients to LDL-C targets, tending to overestimate the magnitude of any effect of doubling the statin dose. LDL-C levels close to goal may contribute to physician reluctance to manage patients more aggressively. Improved understanding of the factors that influence physicians’ treatment recommendations will help guide better guideline implementation and that may overall contribute to better lipid management in clinical practice.

## Methods

### Study design

This questionnaire-based survey was performed in the context of a phase III, two-period, multicenter, double-blind RCT (NCT01154036), published previously [23]. The questionnaire was pre-specified in the study protocol and, along with the RCT, was approved by the institutional review boards at each site (Additional file 3). Written informed consent was obtained from all participants.

In brief, high-risk patients with hypercholesterolemia (either treatment-naïve with LDL-C 166–190 mg/dL, or on stable lipid-lowering therapy with historic lipid values within a range that might reasonably meet the randomization criteria) were enrolled into a 5-week run-in period on atorvastatin 10 mg/day (Figure 5). At the end of the run-in period, patients with LDL-C levels ≥100 and ≤160 mg/dL, and triglyceride levels ≤400 mg/dL, were randomized to ezetimibe 10 mg plus atorvastatin 10 mg, atorvastatin 20 mg, or rosuvastatin 10 mg daily for 6 weeks (period I of the RCT). During period II of the RCT, patients whose LDL-C levels remained elevated at Week 6 changed to a more intensive treatment (Figure 5). This analysis included data only from Period 1.

### Questionnaire

Just prior to randomization into period I of the study i.e., at the randomization visit, physicians were asked to complete a 10-item questionnaire for each patient (Additional file 4). The questionnaire was designed to survey physicians’ pragmatic therapy recommendations for three possible scenarios as if the patient were not enrolled in the RCT and based on the following: (1) LDL-C presumed near goal (between 100–105 mg/dL), (2) LDL-C presumed far from goal (~120 mg/dL), and (3) the observed baseline LDL-C value of each patient on atorvastatin 10 mg, 1 week prior to randomization.

For each scenario, physicians were asked to select from a pre-defined list of treatment choices they would make for each patient (i.e., no change in therapy, double the atorvastatin dose [to 20 mg], add ezetimibe, switch to a different statin, add niacin or fibrates, or other). Multiple choices and write-in choices were also permitted. The questionnaire also asked physicians to specify which patient factors they would consider in deciding their real-life treatment choice(s): LDL-C close to the goal; LDL-C far from goal; desire to achieve more aggressive goal; cardiovascular risk factors; obesity/metabolic syndrome; low high-density lipoprotein cholesterol; high triglyceride; concerns regarding side effects; prior good response to statin; prior poor response to statin; age; sex; patient preference; cost of medication to patient; and reimbursement status. The questionnaire also collected data on patient age, gender, and prior history of CVD, as well as physician characteristics such as type and duration of clinical practice.

Estimated percent change in LDL-C based on the patient’s observed pre-randomization (visit 3; 1 week prior to randomization) LDL-C and the treatment choice recommended by the investigator was compared to the observed percent change in LDL-C following 6 weeks of randomized treatment in period I. Outcomes were defined as the percent change in LDL-C from randomization to Week 6, and the proportion of patients during this period who achieved LDL-C <100 mg/dL or LDL-C <70 mg/dL. The estimated percent change in LDL-C for a recommended treatment choice was calculated from the patient’s pre-randomization (visit 3) LDL-C and the expected incremental change in LDL-C 6 weeks after initiation of the treatment choice recommended by the physician, based on product labeling information and published data (Table 4) [31,45-49]. Estimated outcomes i.e., the proportion of patients achieving LDL-C <100 or <70 mg/dL, were calculated based on the percent incremental benefit that would be expected if the recommended treatment were applied to the observed LDL-C value at visit 3 (1 week prior to randomization) for each patient treated with atorvastatin 10 mg.

**Table 4:** **Expected reductions in LDL-C with atorvastatin 10 mg and the four most common treatment regimens from the questionnaire** [31,45-49]

**Treatment**	Expected LDL-C reduction (%)*	**Estimated additional LDL-C reduction for patients on ATV 10 mg (%)**
ATV 10 mg	38	-
ATV 20 mg	44	10
EZE 10 mg + ATV 10 mg	53	24
EZE + ATV 20 mg	54	26
RSV 10 mg	49	18

*Treatment-naïve patients.

ATV, atorvastatin; EZE, ezetimibe; LDL-C, low-density lipoprotein cholesterol; RSV, rosuvastatin.

### Statistical analysis

All analyses were performed on the all-patients-as-treated population that included all patients randomized into period I and who received at least one dose of study drug.

To identify prognostic clinical factors, physician characteristics, and patient factors that influenced physician treatment choice (as mentioned above), the four most commonly selected treatment recommendations were subject to a pre-specified multivariate forward stepwise regression model. Significant prognostic factors retained from the forward stepwise regression model (at a significance level of 0.15) were analyzed using a univariate approach to obtain the RR and corresponding 95% confidence interval for each prognostic factor for each of the most common treatment choices versus the reference category of no change in therapy. Three separate analyses were conducted for each of the hypothetical and real LDL-C scenarios. An RR >1 indicated that the prognostic factor was more likely to be considered by the physicians in their treatment choice compared to a choice of no change in therapy. Conversely, an RR score <1 suggested the prognostic factor was less likely to be a consideration in selecting a particular treatment, versus no change in therapy.

## Additional files

Additional file 1:
**Association of patient factors with physicians’ treatment recommendations by scenario; stepwise regression relative to no change in therapy, relative risk (95% confidence interval).**
Additional file 2:
**Estimated versus observed treatment outcomes.**
Additional file 3:
**Institutional Review Board.**
Additional file 4:
**Physician pragmatic use questionnaire.**

## Abbreviations

ATV: Atorvastatin
BL: Baseline
CVD: Cardiovascular disease
CI: Confidence interval
EZE: Ezetimibe
LDL-C: Low-density lipoprotein cholesterol
RCT: Randomized controlled trial
RR: Relative risk
RSV: Rosuvastatin
