# Supplementary material for: Patient and physician factors influence decision-making in hypercholesterolemia: a questionnaire-based survey
Source: Lipids Health Dis. 2015 May 19;14:45. doi: 10.1186/s12944-015-0037-y (PMC4457981; doi:10.1186/s12944-015-0037-y)
Supplement: Additional file 1: — Association of patient factors with physicians’ treatment recommendations by scenario; stepwise regression relative to no change in therapy, relative risk (95% confidence interval). [file 12944_2015_37_MOESM1_ESM.docx]

# Additional Files

## Additional File 1 Association of patient factors with physicians’ treatment recommendations by scenario; stepwise regression relative to no change in therapy, relative risk (95% confidence interval)

| **Patient factor** | **LDL-C close to goal (100–105 mg/dL)** | | | **LDL-C far from goal (~120 mg/dL)** | | | **Observed baseline LDL-C** | | |
| --- | --- | --- | --- | --- | --- | --- | --- | --- | --- |
|  | **Double ATV dose (n = 656)** | **Add EZE (n = 133)** | **Double ATV dose *and* add EZE (n = 31)** | **Double ATV dose (n = 799)** | **Add EZE (n = 239)** | **Double ATV dose *and* add EZE (n = 178)** | **Double ATV dose (n = 712)** | **Add EZE (n = 230)** | **Double ATV dose *and* add EZE (n = 142)** |
| Prior poor response to statin | 0.63  (0.44–0.90) | 0.71  (0.37–1.33) | 0.30 (0.04–2.11) | 0.53 (0.37–0.76) | 0.56 (0.36–0.87) | 0.60  0.38–0.96) | 1.62 (1.02–2.58) | 3.26 (2.02–5.27) | 2.45 (1.42–4.24) |
| Prior good response to statin | 0.66  (0.54–0.80) | 0.18  (0.09–0.37) | 0.65 (0.31–1.35) | 0.88 (0.58–1.33) | 0.29 (0.15–0.56) | 0.87 (0.53–1.44) | 0.82 (0.63–1.07) | 0.17 (0.08–0.33) | 0.51 (0.32–0.84) |
| Concerns about side effects | 0.42  (0.29–0.59) | 1.11 (0.72–1.69) | 1.29 (0.62–2.71) | 0.32 (0.22–0.48) | 0.35  (0.21–0.59) | 0.54 (0.33–0.88) | 0.28 (0.19–0.42) | 0.58 (0.38–0.90) | 0.51 (0.29–0.88) |
| Age | 0.71  (0.57–0.88) | 0.46 (0.28–0.75) | 1.05  (0.57–1.94) | - | - | - | - | - | - |
| Gender | - | - | - | 0.67 (0.46–0.98) | 0.38 (0.23–0.65) | 0.82 (0.52–1.30) | 0.76 (0.55–1.04) | 0.51 (0.31–0.83) | 1.18 (0.78–1.77) |
| Cardiovascular risk factors | 1.47  (1.34–1.60) | 1.38 (1.20–1.57) | 1.07 (0.77–1.48) | 1.00 (0.87–1.15) | 0.97 (0.83–1.14) | 1.21  (1.04–1.40) | 1.48 (1.29–1.71) | 1.50 (1.27–1.76) | 1.71 (1.46–2.01) |
| LDL-C close to goal | 0.75  (0.71–0.79) | 0.72  (0.63–0.82) | 0.86 (0.71–1.04) | - | - | - | 0.52  (0.47–0.57) | 0.46 (0.39–0.54) | 0.27 (0.20–0.36) |
| LDL-C far from goal | - | - | - | 1.23 (1.05–1.44) | 1.26 (1.06–1.49) | 1.48 (1.26–1.75) | 7.61 (4.44–13.05) | 10.04 (5.81–17.34) | 16.70 (9.77–28.54) |
| Obesity/metabolic syndrome | 1.45  (1.19–1.78) | 1.01 (0.68–1.48) | 2.25 (1.44–3.50) | - | - | - | - | - | - |
| High triglyceride | - | - | - | 0.62 (0.35–1.08) | 1.19 (0.66–2.14) | 1.34 (0.74–2.44) | 1.09 (0.66–1.80) | 1.66 (0.95–2.90) | 1.89 (1.03–3.45) |
| Cost of medication | 0.96 (0.77–1.21) | 0.32 (0.16–0.64) | 0.69 (0.27–1.75) | - | - | - | - | - | - |
| Reimbursement status | 0.84 (0.55–1.27) | 0.32 (0.10–1.01) | 2.27 (0.97–5.31) | 3.94 (0.98–15.86) | 0.63 (0.11–3.70) | 6.18 (1.48–35.74) | 1.16 (0.64–2.08) | 0.33 (0.11–1.00) | 1.75 (0.85–3.63) |
| Desire to achieve more aggressive goal | 4.16 (3.35–5.18) | 3.53 (2.66–4.68) | 4.18 (2.81–6.22) | 2.23  (1.51–3.28) | 2.19 (1.46–3.28) | 2.78 (1.87–4.15) | 4.21 (3.00–5.92) | 3.27 (2.26–4.74) | 5.24 (3.67–7.48) |

Multinominal logistic regression model of physician treatment choice, with no change in therapy as reference category (LDL-C close to goal, n=648; LDL-C far from goal, n=100; observed baseline LDL-C, n=268). RR >1: prognostic factor more likely to be considered by the physician in their treatment choice compared to a choice of ‘no change in therapy’; RR <1: prognostic factor less likely to be considered versus no change in therapy.

Blank cells, factor not selected by the forward stepwise regression model as a prognostic factor for the relevant scenario.

ATV, atorvastatin; EZE, ezetimibe; LDL-C, low-density lipoprotein cholesterol; RR, relative risk
