# Supplementary material for: Patient and physician factors influence decision-making in hypercholesterolemia: a questionnaire-based survey
Source: Lipids Health Dis. 2015 May 19;14:45. doi: 10.1186/s12944-015-0037-y (PMC4457981; doi:10.1186/s12944-015-0037-y)
Supplement: Additional file 2: — Estimated versus observed treatment outcomes. [file 12944_2015_37_MOESM2_ESM.doc]

Additional File 2 Estimated versus observed treatment outcomes

| **Physicians’ recommendation (estimated)* Randomized treatment (observed)^†^** | **RCT baseline LDL-C (mg/dL)** | **% change from baseline in  LDL-C at week 6** | **Patients achieving treatment goal (%)** | |
| --- | --- | --- | --- | --- |
|  |  |  | **<100 mg/dL** | **<70 mg/dL** |
| **No change in therapy (estimated), n = 260** | 111.4 | 0 | 0.4 | 0.0 |
| EZE 10 + ATV 10 (observed), n = 18 | 111.5 | –20.3 | 66.7 | 33.3 |
| ATV 20 (observed), n = 98 | 110.7 | –1.1 | 44.9 | 4.1 |
| RSV 10 (observed), n = 144 | 111.9 | –6.4 | 50.7 | 6.9 |
| **Double ATV dose (estimated), n = 698** | 119.2 | –10.0 | 30.1 | 0.0 |
| EZE 10 + ATV 10 (observed), n = 54 | 119.3 | –20.8 | 59.3 | 20.4 |
| ATV 20 (observed), n = 220 | 119.4 | –11.4 | 41.4 | 3.2 |
| RSV 10 (observed), n = 424 | 119.1 | –12.4 | 45.0 | 7.5 |
| **Add EZE (estimated), n = 219** | 123.7 | –24.0 | 65.3 | 0.0 |
| EZE 10 + ATV 10 (observed), n = 19 | 122.9 | –24.0 | 52.6 | 26.3 |
| ATV 20 (observed), n = 66 | 124.4 | –9.3 | 28.8 | 1.5 |
| RSV 10 (observed), n = 134 | 123.5 | –12.5 | 40.3 | 5.2 |
| **Double ATV dose *and* add EZE (estimated), n = 140** | 129.3 | –26.0 | 57.1 | 0.0 |
| EZE 10 + ATV 10 (observed), n = 14 | 134.0 | –15.4 | 35.7 | 0.0 |
| ATV 20 (observed), n = 43 | 123.4 | –8.5 | 27.9 | 2.3 |
| RSV 10 (observed), n = 83 | 131.5 | –13.0 | 30.1 | 6.0 |
| Switch to RSV 10 (estimated), n = 40 | 124.8 | –18.0 | 42.5 | 0.0 |
| EZE 10 + ATV 10 (observed), n = 1 | 136.0 | –39.2 | 100.0 | 0.0 |
| ATV 20 (observed), n = 10 | 122.5 | –1.4 | 20.0 | 0.0 |
| RSV 10 (observed), n = 29 | 125.2 | –10.6 | 31.0 | 6.9 |

Values shown are mean values.

*****Estimated outcomes of physician recommended treatments were based on data for treatment-naïve patients in product labels and literature.

^†^Observed (RCT) outcomes at end of period I for randomized treatment.

Estimated (estimated) outcomes calculated based on % incremental benefit expected if the recommended treatment were applied to the observed LDL-C value at the end of the run-in phase for each patient treated with atorvastatin 10 mg; individual estimates were averaged across all patients where a particular treatment was recommended.

The proportion of patients achieving a treatment goal (<100 or <70 mg/dL) was derived by applying each patients’ estimated LDL-C change associated with the recommended treatment.

ATV, atorvastatin; EZE, ezetimibe; RCT, randomized controlled trial; RSV, rosuvastatin.
