# Supplementary material for: Patient and physician factors influence decision-making in hypercholesterolemia: a questionnaire-based survey
Source: Lipids Health Dis. 2015 May 19;14:45. doi: 10.1186/s12944-015-0037-y (PMC4457981; doi:10.1186/s12944-015-0037-y)
Supplement: Additional file 3: — Institutional Review Board. [file 12944_2015_37_MOESM3_ESM.docx]

| **Protocol Study Number/ Site Number** | **Name and Address of Independent Ethics Committee (IEC)** |  |
| --- | --- | --- |
| 162/0001 | Comité voor Medische Ehiek - Algemeen Ziekenhuis KLINA  Augustijnslei 100  2930 Brasschaat Belgium |  |
| 162/0002 | Universitair Ziekenhuis Antwerpen - Secretariaat Ethisch Comité Wilrijkstraat 10  2650 Edegem Belgium |  |
| 162/0003 | CPP Est 1  7 boulevard Jeanne d'Arc  Faculté de Médecine - 4ème étage 21079 Dijon cedex  France |  |
| 162/0004 | CPP Est 1  7 boulevard Jeanne d'Arc  Faculté de Médecine - 4ème étage 21079 Dijon cedex  France |  |
| 162/0005 | CPP Est 1  7 boulevard Jeanne d'Arc  Faculté de Médecine - 4ème étage 21079 Dijon cedex  France |  |
| 162/0006 | CPP Est 1  7 boulevard Jeanne d'Arc  Faculté de Médecine - 4ème étage 21079 Dijon cedex  France |  |
| 162/0007 | CPP Est 1  7 boulevard Jeanne d'Arc  Faculté de Médecine - 4ème étage  21079 Dijon cedex France |  |
| 162/0008 | CPP Est 1  7 boulevard Jeanne d'Arc  Faculté de Médecine - 4ème étage 21079 Dijon cedex  France |  |
| 162/0009 | CPP Est 1  7 boulevard Jeanne d'Arc  Faculté de Médecine - 4ème étage 21079 Dijon cedex  France |  |
| 162/0010 | Lithuanian Bioethics Committee Didzioji str. 22  LT-01128 Vilnius |  |
| 162/0011 | Lithuanian Bioethics Committee Didzioji str. 22  LT-01128 Vilnius |  |
| 162/0012 | Lithuanian Bioethics Committee Didzioji str. 22  LT-01128 Vilnius |  |
| 162/0013 | Lithuanian Bioethics Committee Didzioji str. 22  LT-01128 Vilnius |  |
| 162/0014 | Lithuanian Bioethics Committee Didzioji str. 22  LT-01128 Vilnius |  |
| 162/0015 | Lithuanian Bioethics Committee Didzioji str. 22  LT-01128 Vilnius |  |
| 162/0016 | Lithuanian Bioethics Committee Didzioji str. 22  LT-01128 Vilnius |  |
| 162/0017 | Lithuanian Bioethics Committee Didzioji str. 22  LT-01128 Vilnius | |
| 162/0019 | Hospital Vall d'Hebrón  Dirección de investigación, planta 2 - Edifici Recerca  Passeig de la Vall d'Hebrón s/n 08035 Barcelona  Spain | |
| 162/0020 | Hospital Vall d'Hebrón  Dirección de investigación, planta 2 - Edifici Recerca  Passeig de la Vall d'Hebrón s/n 08035 Barcelona  Spain | |
| 162/0021 | Hospital Vall d'Hebrón  Dirección de investigación, planta 2 - Edifici Recerca  Passeig de la Vall d'Hebrón s/n 08035 Barcelona  Spain | |
| 162/0022 | Hospital Vall d'Hebrón  Dirección de investigación, planta 2 - Edifici Recerca  Passeig de la Vall d'Hebrón s/n 08035 Barcelona  Spain | |
| 162/0023 | Hospital Vall d'Hebrón  Dirección de investigación, planta 2 - Edifici Recerca  Passeig de la Vall d'Hebrón s/n 08035 Barcelona  Spain | |
| 162/0024 | Hospital Vall d'Hebrón  Dirección de investigación, planta 2 - Edifici Recerca  Passeig de la Vall d'Hebrón s/n 08035 Barcelona  Spain | |
| 162/0025 | Hospital Vall d'Hebrón  Dirección de investigación, planta 2 - Edifici Recerca  Passeig de la Vall d'Hebrón s/n 08035 Barcelona  Spain | |
| 162/0026 | NRES Committee South Central Southampton A, South West REC Centre, Level 3, Block B, Whitefriars,  Bristol BS1 2NT United Kingdom | |
| 162/0027 | NRES Committee South Central Southampton A, South West REC Centre, Level 3, Block B, Whitefriars,  Bristol BS1 2NT United Kingdom | |
| 162/0028 | NRES Committee South Central Southampton A, South West REC Centre, Level 3, Block B, Whitefriars,  Bristol BS1 2NT United Kingdom | |
| 162/0029 | NRES Committee South Central Southampton A, South West REC Centre, Level 3, Block B, Whitefriars,  Bristol BS1 2NT United Kingdom | |
| 162/0030 | NRES Committee South Central Southampton A, South West REC Centre, Level 3, Block B, Whitefriars,  Bristol BS1 2NT United Kingdom | |
| 162/0031 | NRES Committee South Central Southampton A, South West REC Centre, Level 3, Block B, Whitefriars,  Bristol BS1 2NT United Kingdom | |
| 162/0032 | NRES Committee South Central Southampton A, South West REC Centre, Level 3, Block B, Whitefriars,  Bristol BS1 2NT United Kingdom | |
| 162/0033 | NRES Committee South Central Southampton A, South West REC Centre, Level 3, Block B, Whitefriars,  Bristol BS1 2NT United Kingdom | |
| 162/0034 | NRES Committee South Central Southampton A, South West REC Centre, Level 3, Block B, Whitefriars,  Bristol BS1 2NT United Kingdom | |
| 162/0035 | NRES Committee South Central Southampton A, South West REC Centre, Level 3, Block B, Whitefriars,  Bristol BS1 2NT United Kingdom | |
| 162/0036 | NRES Committee South Central Southampton A, South West REC Centre, Level 3, Block B, Whitefriars,  Bristol BS1 2NT  United Kingdom | |
| 162/0038 | NRES Committee South Central Southampton A, South West REC Centre, Level 3, Block B, Whitefriars,  Bristol BS1 2NT United Kingdom | |
| 162/0039 | KOMISIJA REPUBLIKE SLOVENIJE ZA MEDICINSKO ETIKO - NATIONAL MEDICAL ETHICS COMMITTEE  University Institute of Clinical Neurophysiology,Zaloška c. 7 SI-1525 Ljubljana | |
| 162/0040 | KOMISIJA REPUBLIKE SLOVENIJE ZA MEDICINSKO ETIKO - NATIONAL MEDICAL ETHICS COMMITTEE  University Institute of Clinical Neurophysiology,Zaloška c. 7 SI-1525 Ljubljana | |
| 162/0041 | KOMISIJA REPUBLIKE SLOVENIJE ZA MEDICINSKO ETIKO - NATIONAL MEDICAL ETHICS COMMITTEE  University Institute of Clinical Neurophysiology,Zaloška c. 7 SI-1525 Ljubljana | |
| 162/0042 | Komisja Bioetyczna przy Okregowej Izbie Lekarskiej  ul. Sniadeckich 33  80-204 Gdansk Poland | |
| 162/0043 | Komisja Bioetyczna przy Okregowej Izbie Lekarskiej  ul. Sniadeckich 33  80-204 Gdansk  Poland | |
| 162/0045 | Komisja Bioetyczna przy Okregowej Izbie Lekarskiej  ul. Sniadeckich 33  80-204 Gdansk Poland | |
| 162/0046 | Komisja Bioetyczna przy Okregowej Izbie Lekarskiej  ul. Sniadeckich 33  80-204 Gdansk Poland | |
| 162/0047 | Komisja Bioetyczna przy Okregowej Izbie Lekarskiej  ul. Sniadeckich 33  80-204 Gdansk Poland | |
| 162/0048 | Komisja Bioetyczna przy Okregowej Izbie Lekarskiej  ul. Sniadeckich 33  80-204 Gdansk Poland | |
| 162/0049 | Komisja Bioetyczna przy Okregowej Izbie Lekarskiej  ul. Sniadeckich 33  80-204 Gdansk Poland | |
| 162/0050 | Rambam Medical Campus POBox 9602  8, Ha'aliya St Bat Galim 31096 Haifa  Israel | |
| 162/0051 | Meir Hospital  59 Tchernihovski St.  44281 Kfar Saba Israel | |
| 162/0052 | Sheba Medical Center Tel Hashomer  52621 Ramat Gan Israel | |
| 162/0053 | Haemek Medical Center Rabin Boulevard  18101 Afula Israel | |
| 162/0054 | Kaplan Medical Center  P.O.B. 1 76100 Rehovot Israel | |
| 162/0055 | Bnai Zion Golomb 47  St P.O.B. 4947  31048 Haifa Israel | |
| 162/0056 | Rebecca Seiff Medical Center 13100 Safed  Israel | |
| 162/0057 | Edith Wolfson Medical Center 62 Halochamim Street  58100 Holon Israel | |
| 162/0058 | Meir Hospital  59 Tchernihovski St.  44281 Kfar Saba Israel | |
| 162/0059 | Tel Aviv Medical Center Weitzman St 6  64239 Tel Aviv  Israel | |
| 162/0060 | Holy Family Hospital 16100 Nazareth Israel | |
| 162/0061 | Rabin Medical Center Campus Beilinson Jabotinski 39  St Petach 49100 Tikva Israel | |
| 162/0062 | Sisätautien eettinen toimikunta Tutkimuseettiset toimikunnat (Medisiininen eettinen toimikunta - name of the CEC during the summer period only) Biomedicum Helsinki 2 C, 7. kerros  Tukholmankatu 8 C, PL 705  00029 HUS Helsinki Finland | |
| 162/0063 | Sisätautien eettinen toimikunta Tutkimuseettiset toimikunnat (Medisiininen eettinen toimikunta - name of the CEC during the summer period only) Biomedicum Helsinki 2 C, 7. kerros  Tukholmankatu 8 C, PL 705  00029 HUS Helsinki Finland | |
| 162/0064 | Sisätautien eettinen toimikunta Tutkimuseettiset toimikunnat (Medisiininen eettinen toimikunta - name of the CEC during the summer period only) Biomedicum Helsinki 2 C, 7. kerros  Tukholmankatu 8 C, PL 705  00029 HUS Helsinki  Finland | |
| 162/0065 | Sisätautien eettinen toimikunta Tutkimuseettiset toimikunnat (Medisiininen eettinen toimikunta - name of the CEC during the summer period only) Biomedicum Helsinki 2 C, 7. kerros  Tukholmankatu 8 C, PL 705  00029 HUS Helsinki Finland | |
| 162/0067 | Sisätautien eettinen toimikunta Tutkimuseettiset toimikunnat (Medisiininen eettinen toimikunta - name of the CEC during the summer period only) Biomedicum Helsinki 2 C, 7. kerros  Tukholmankatu 8 C, PL 705  00029 HUS Helsinki Finland | |
| 162/0068 | Hospital Vall d'Hebrón  Dirección de investigación, planta 2 - Edifici Recerca  Passeig de la Vall d'Hebrón s/n 08035 Barcelona  Spain | |
| 162/0091 | CEIC – Comissão de Ética para a Investigação Clínica  Parque da Saúde de Lisboa Av. do Brasil, 53 - Pav. 17-A 1749-004 Lisboa  Portugal | |
| 162/0092 | CEIC – Comissão de Ética para a Investigação Clínica  Parque da Saúde de Lisboa Av. do Brasil, 53 - Pav. 17-A  1749-004 Lisboa Portugal | |
| 162/0093 | CEIC – Comissão de Ética para a Investigação Clínica  Parque da Saúde de Lisboa Av. do Brasil, 53 - Pav. 17-A 1749-004 Lisboa  Portugal | |
| 162/0094 | CEIC – Comissão de Ética para a Investigação Clínica  Parque da Saúde de Lisboa Av. do Brasil, 53 - Pav. 17-A 1749-004 Lisboa  Portugal | |
| 162/0095 | Comisia Nationala de Etica pentru Studiul Clinic al Medicamentului str. Av. Sanatescu nr. 48, sector 1  011478 Bucharest Romania | |
| 162/0096 | Comisia Nationala de Etica pentru Studiul Clinic al Medicamentului str. Av. Sanatescu nr. 48, sector 1  011478 Bucharest Romania | |
| 162/0097 | Comisia Nationala de Etica pentru Studiul Clinic al Medicamentului str. Av. Sanatescu nr. 48, sector 1  011478 Bucharest Romania | |
| 162/0098 | Comisia Nationala de Etica pentru Studiul Clinic al Medicamentului str. Av. Sanatescu nr. 48, sector 1  011478 Bucharest  Romania | |
| 162/0099 | Comisia Nationala de Etica pentru Studiul Clinic al Medicamentului str. Av. Sanatescu nr. 48, sector 1  011478 Bucharest Romania | |
| 162/0100 | Comisia Nationala de Etica pentru Studiul Clinic al Medicamentului str. Av. Sanatescu nr. 48, sector 1  011478 Bucharest Romania | |
| 162/0101 | Comisia Nationala de Etica pentru Studiul Clinic al Medicamentului str. Av. Sanatescu nr. 48, sector 1  011478 Bucharest Romania | |
| 162/0102 | Comisia Nationala de Etica pentru Studiul Clinic al Medicamentului str. Av. Sanatescu nr. 48, sector 1  011478 Bucharest Romania | |
| 162/0103 | Comisia Nationala de Etica pentru Studiul Clinic al Medicamentului str. Av. Sanatescu nr. 48, sector 1  011478 Bucharest Romania | |
| 162/0104 | Comisia Nationala de Etica pentru Studiul Clinic al Medicamentului str. Av. Sanatescu nr. 48, sector 1  011478 Bucharest Romania | |
| 162/0105 | Comisia Nationala de Etica pentru Studiul Clinic al Medicamentului str. Av. Sanatescu  nr. 48, sector 1  011478 Bucharest Romania | |
| 162/0106 | Comisia Nationala de Etica pentru Studiul Clinic al Medicamentului str. Av. Sanatescu nr. 48, sector 1  011478 Bucharest Romania | |
| 162/0107 | Comité de Etica Independiente en Investigación Clínica  Dr Carlos A Barclay Larrea 1381 - 3° A C1117ABK Buenos Aires Argentina  Comité de Docencia e Investigación, CER San Juan  Laprida 568 E  5400 San Juan Argentina | |
| 162/0108 | Comité de Etica del Hospital Italiano Regional del Sur Necochea 675 Bahia Blanca  Buenos Aires Argentina  Comité etica de la Investigacion Hospital Italiano de La plata -Hospital Italiano de La Plata  Avenida 51 N° 1725 La Plata  B1900AXI Buenos Aires Argentina | |
| 162/0109 | Comité de Etica del Hospital Italiano Regional del Sur Necochea 675 Bahia Blanca  Buenos Aires Argentina  Comité de Docencia e Investigación, Hospital Italiano de La Plata  Karina A. Cricri  Avenida 51 N° 1725 La Plata B1900AXI Buenos Aires Argentina | |
| 162/0110 | Comité de Etica Independiente en Investigación Clínica  Dr Carlos A Barclay Larrea 1381 - 3° A C1117ABK Buenos Aires Argentina  Comité de Docencia e Investigación Hospital San Bernardo  69 Salta 4406CLA  Argentina | |
| 162/0111 | Comité de Etica Independiente en Investigación Clínica  Dr Carlos A Barclay Larrea 1381 - 3° A C1117ABK Buenos Aires Argentina  Comité de Docencia e Investigación Instituto de Cardiología de Corrientes  Simón Bolívar N° 1334 Corrientes  Argentina | |
| 162/0112 | Comité de Bioética del Instituto de Investigaciones Clínicas de Rosario Paraguay 160  S2000CVD Santa Fe Argentina | |
| 162/0113 | Comité Independiente de Etica en Investigación Clínica en Seres Humanos Fundación Rusculleda Av. Colón 2057 X5003DCE Córdoba  Argentina | |
| 162/0114 | Comité Independiente de Etica en Investigación Clínica en Seres Humanos Fundación Rusculleda Av. Colón 2057 X5003DCE Córdoba  Argentina | |
| 162/0115 | Comité de Ética en Investigacion Instituto de Investigaciones Clinicas  Av. Colon 3364 Mar del Plata  B7600FZN Buenos Aires Argentina | |
| 162/0116 | Comité de Etica Independiente en Investigación Clínica  Dr Carlos A Barclay Larrea 1381 - 3° A C1117ABK Buenos Aires Argentina | |
| 162/0117 | Comité de Etica Independiente en Investigación Clínica  Dr Carlos A Barclay  Larrea 1381 - 3° A C1117ABK Buenos Aires Argentina  Comité de Docencia e Investigación - CEDIR  San Martin 3169  3000 Santa Fe Argentina | |
| 162/0118 | Comité de Ética en Investigacion Instituto de Investigaciones Clinicas  Av. Colon 3364 Mar del Plata  B7600FZN Buenos Aires Argentina  Comité de Docencia e Investigación ( DIM Clinica Privada Belgrano 136  B1704ETD Ramos Mejía Argentina) | |
| 162/0119 | Conjoint Health Research Ethics Board Canada | |
| 162/0120 | IRB Services Hollandview Trail 372  Suite 300  L4G 0A5 Aurora Canada | |
| 162/0121 | IRB Services Hollandview Trail 372  Suite 300  L4G 0A5 Aurora Canada | |
| 162/0122 | IRB Services  Hollandview Trail 372  Suite 300  L4G 0A5 Aurora Canada | |
| 162/0123 | IRB Services Hollandview Trail 372  Suite 300  L4G 0A5 Aurora Canada | |
| 162/0125 | IRB Services Hollandview Trail 372  Suite 300  L4G 0A5 Aurora Canada | |
| 162/0126 | IRB Services Hollandview Trail 372  Suite 300  L4G 0A5 Aurora Canada | |
| 162/0127 | IRB Services Hollandview Trail 372  Suite 300  L4G 0A5 Aurora Canada | |
| 162/0128 | IRB Services Hollandview Trail 372  Suite 300  L4G 0A5 Aurora Canada | |
| 162/0132 | IRB Services Hollandview Trail 372  Suite 300  L4G 0A5 Aurora  Canada | |
| 162/0133 | IRB Services Hollandview Trail 372  Suite 300  L4G 0A5 Aurora Canada | |
| 162/0135 | IRB Services Hollandview Trail 372  Suite 300  L4G 0A5 Aurora Canada | |
| 162/0137 | IRB Services Hollandview Trail 372  Suite 300  L4G 0A5 Aurora Canada | |
| 162/0139 | IRB Services Hollandview Trail 372  Suite 300  L4G 0A5 Aurora Canada | |
| 162/0146 | Comite Etico cientifico del Servicio de Salud Araucania Sur Andres Bello #636  4791301 Temuco Chile | |
| 162/0147 | Comite Etico Cientifico del Servicio de Salud Metropolitano Oriente  Avenue Salvador #364  7500922 Providencia Santiago Chile | |
| 162/0148 | Comite Etico Cientifico del Servicio de Salud Metropolitano Oriente  Avenue Salvador #364  7500922 Providencia Santiago Chile | |
| 162/0149 | Comite Etico cientifico del Servicio de Salud Araucania Sur Andres Bello #636  4791301 Temuco Chile | |
| 162/0150 | Comite Etico cientifico del Servicio de salud Metropolitano Central  Avenue Santa Rosa 1234 8360160 Santiago  Chile | |
| 162/0151 | Comite Etico Cientifico del Servicio de Salud Metropolitano Oriente  Avenue Salvador #364  7500922 Providencia Santiago Chile | |
| 162/0152 | CIRCIE  Colombia | |
| 162/0154 | Comité de Etica de la Investigación de CEMDE  Colombia | |
| 162/0155 | Comité de Etica en Investigación de la FOSCAL  Av El Bosque No 23 - 60 Autopista a Floridablanca Bucaramanga  Colombia | |
| 162/0156 | Comité de Etica en la Investigación - Clínica CAIMED  Carrera 42 A No. 17 - 50 Bogotá  Colombia | |
| 162/0162 | Hospital Vall d'Hebrón  Dirección de investigación, planta 2 - Edifici Recerca  Passeig de la Vall d'Hebrón s/n 08035 Barcelona  Spain | |
| 162/0163 | Ethikkommission der Landesärztekammer Hessen  Im Vogelsgesang 3 60488 Frankfurt a. M Germany | |
| 162/0164 | Ethikkommission der Landesärztekammer Hessen  Im Vogelsgesang 3 60488 Frankfurt a. M Germany | |
| 162/0165 | Ethikkommission der Landesärztekammer Hessen  Im Vogelsgesang 3 60488 Frankfurt a. M Germany | |
| 162/0166 | Ethikkommission der Landesärztekammer Hessen  Im Vogelsgesang 3 60488 Frankfurt a. M Germany | |
| 162/0167 | Ethikkommission der Landesärztekammer Hessen  Im Vogelsgesang 3 Frankfurt a. M 60488 | |
| 162/0168 | Ethikkommission der Landesärztekammer Hessen  Im Vogelsgesang 3 60488 Frankfurt a. M Germany | |
| 162/0169 | Ethikkommission der Landesärztekammer Hessen  Im Vogelsgesang 3 60488 Frankfurt a. M Germany | |
| 162/0170 | Tallinn Medical Research Ethics Committee National Institute for Health Development Hiiu 42  11619 Tallinn Finland | |
| 162/0171 | Tallinn Medical Research Ethics Committee National Institute for Health Development Hiiu 42  11619 Tallinn Finland | |
| 162/0172 | Tallinn Medical Research Ethics Committee National Institute for Health Development Hiiu 42  11619 Tallinn Finland | |
| 162/0173 | Tallinn Medical Research Ethics Committee National Institute for Health Development Hiiu 42  11619 Tallinn Finland | |
| 162/0174 | Ethics Committee for Multicenter Trials Sveta nedelya Sq 5  1000 Sofia Bulgaria | |
| 162/0175 | Ethics Committee for Multicenter Trials Sveta nedelya Sq 5  1000 Sofia Bulgaria | |
| 162/0177 | Ethics Committee for Multicenter Trials Sveta nedelya Sq 5  1000 Sofia Bulgaria | |
| 162/0179 | Ethics Committee for Multicenter Trials Sveta nedelya Sq 5  1000 Sofia Bulgaria | |
| 162/0180 | Ethics Committee for Multicenter Trials Sveta nedelya Sq 5  1000 Sofia Bulgaria | |
| 162/0182 | Ethics Committee for Multicenter Trials Sveta nedelya Sq 5  1000 Sofia Bulgaria | |
| 162/0183 | Ethics Committee for Multicenter Trials Sveta nedelya Sq 5  1000 Sofia Bulgaria | |
| 162/0184 | Ethics Committee for Multicenter Trials Sveta nedelya Sq 5  1000 Sofia Bulgaria | |
| 162/0185 | Ethics Committee for Multicenter Trials Sveta nedelya Sq 5  1000 Sofia Bulgaria | |
| 162/0186 | Ethics Committee for Multicenter Trials Sveta nedelya Sq 5  1000 Sofia Bulgaria | |
| 162/0187 | Ethics Committee for Multicenter Trials Sveta nedelya Sq 5  1000 Sofia Bulgaria | |
| 162/0189 | Etická komise FN a LF UP Olomouc  I. P. Pavlova 6 775 20 Olomouc Czech Republic | |
| 162/0191 | Etická komise FN a LF UP Olomouc  I. P. Pavlova 6 775 20 Olomouc Czech Republic | |
| 162/0192 | Etická komise FN a LF UP Olomouc  I. P. Pavlova 6 775 20 Olomouc Czech Republic  Na Homolce Hospital (Nemocnice Na Homolce)  Roentgenova 2  150 19 Praha 5 Czech Republic | |
| 162/0193 | Etická komise FN a LF UP Olomouc  I. P. Pavlova 6 775 20 Olomouc Czech Republic  Lokalni eticka komise ResTrial s.r.o. V sadech 15/4  Bubencec  160 00 Praha 6 Czech Republic | |
| 162/0194 | Etická komise FN a LF UP Olomouc  I. P. Pavlova 6 775 20 Olomouc Czech Republic  St. Anne's University Hospital Brno (FN u sv. Anny v Brně)  Pekařská 53  656 91 Brno Czech Republic | |
| 162/0195 | Etická komise FN a LF UP Olomouc  I. P. Pavlova 6 775 20 Olomouc Czech Republic | |
| 162/0196 | Etická komise FN a LF UP Olomouc  I. P. Pavlova 6 775 20 Olomouc Czech Republic | |
| 162/0197 | Etická komise FN a LF UP Olomouc  I. P. Pavlova 6 775 20 Olomouc Czech Republic | |
| 162/0198 | Etická komise FN a LF UP Olomouc  I. P. Pavlova 6 775 20 Olomouc Czech Republic | |
| 162/0199 | Etická komise FN a LF UP Olomouc  I. P. Pavlova 6 775 20 Olomouc Czech Republic | |
| 162/0200 | Etická komise FN a LF UP Olomouc  I. P. Pavlova 6 775 20 Olomouc Czech Republic | |
| 162/0201 | Etická komise FN a LF UP Olomouc  I. P. Pavlova 6 775 20 Olomouc Czech Republic | |
| 162/0202 | Etická komise FN a LF UP Olomouc  I. P. Pavlova 6 775 20 Olomouc Czech Republic | |
| 162/0203 | Etická komise FN a LF UP Olomouc  I. P. Pavlova 6 775 20 Olomouc Czech Republic | |
| 162/0204 | Sredisnje Eticko Povjerenstvo Ksaverska cesta 4  10000 Zagreb Croatia | |
| 162/0206 | Sredisnje Eticko Povjerenstvo Ksaverska cesta 4  10000 Zagreb Croatia | |
| 162/0207 | Sredisnje Eticko Povjerenstvo Ksaverska cesta 4  10000 Zagreb Croatia | |
| 162/0208 | Den Videnskabsetiske Komité for Region Syddanmark  Regionshuset Damhaven 12  7100 Vejle Denmark | |
| 162/0209 | Den Videnskabsetiske Komité for Region Syddanmark  Regionshuset Damhaven 12  7100 Vejle Denmark | |
| 162/0210 | Den Videnskabsetiske Komité for Region Syddanmark  Regionshuset Damhaven 12  7100 Vejle Denmark | |
| 162/0211 | Den Videnskabsetiske Komité for Region Syddanmark  Regionshuset Damhaven 12  7100 Vejle Denmark | |
| 162/0212 | Den Videnskabsetiske Komité for Region Syddanmark  Regionshuset Damhaven 12  7100 Vejle Denmark | |
| 162/0213 | Egészségügyi Tudományos Tanács Klinikai Farmakológiai Etikai Bizottsága  Arany János u. 6-8. 1051 Budapest Hungary | |
| 162/0214 | Egészségügyi Tudományos Tanács Klinikai Farmakológiai Etikai Bizottsága  Arany János u. 6-8. 1051 Budapest Hungary | |
| 162/0215 | Egészségügyi Tudományos Tanács Klinikai  Farmakológiai Etikai Bizottsága Arany János u. 6-8.  1051 Budapest Hungary | |
| 162/0217 | Egészségügyi Tudományos Tanács Klinikai Farmakológiai Etikai Bizottsága  Arany János u. 6-8. 1051 Budapest Hungary | |
| 162/0218 | Egészségügyi Tudományos Tanács Klinikai Farmakológiai Etikai Bizottsága  Arany János u. 6-8. 1051 Budapest Hungary | |
| 162/0220 | Egészségügyi Tudományos Tanács Klinikai Farmakológiai Etikai Bizottsága  Arany János u. 6-8. 1051 Budapest Hungary | |
| 162/0221 | Egészségügyi Tudományos Tanács Klinikai Farmakológiai Etikai Bizottsága  Arany János u. 6-8. 1051 Budapest Hungary | |
| 162/0222 | Egészségügyi Tudományos Tanács Klinikai Farmakológiai Etikai Bizottsága  Arany János u. 6-8. 1051 Budapest Hungary | |
| 162/0223 | Egészségügyi Tudományos Tanács Klinikai Farmakológiai Etikai Bizottsága  Arany János u. 6-8. 1051 Budapest  Hungary | |
| 162/0224 | Egészségügyi Tudományos Tanács Klinikai Farmakológiai Etikai Bizottsága  Arany János u. 6-8. 1051 Budapest Hungary | |
| 162/0225 | Egészségügyi Tudományos Tanács Klinikai Farmakológiai Etikai Bizottsága  Arany János u. 6-8. 1051 Budapest Hungary | |
| 162/0226 | Comitato di Bioetica della Fondazione IRCCS Policlinico S. Matteo di Pavia Viale Golgi 19  27100 Pavia Italy | |
| 162/0227 | Comitato Etico dell’Azienda Ospedaliero Universitaria Policlinico Vittorio Emanuele di Catania  Via S. Sofia 78 95123 Catania Italy | |
| 162/0228 | Comitato Di Bioetica della ASL di Sassari Via Monte Grappa 82  07100 Sassari Italy | |
| 162/0229 | Comitato Etico-Scientifico dell’Azienda Ospedaliera Ospedale Niguarda Ca´ Granda di Milano  Piazza Ospedale Maggiore 3 20162 Milano  Italy | |
| 162/0230 | Comitato Etico dell'Azienda Ospedaliera Universitaria Policlinico Paolo Giaccone dell'Università' degli Studi di Palermo Via Del Vespro 127  90129 Palermo Italy | |
| 162/0231 | Comitato Etico per la Sperimentazione Clinica della Provincia di Treviso Borgo Cavalli 42  31100 Treviso Italy | |
| 162/0232 | Comitato Etico dell'Universita' degli Studi Gabriele D`Annunzio e della ASL 2 Lanciano-Vasto- Chieti di Chieti  Via dei Vestini 31 66013 Chieti  Italy | |
| 162/0233 | Regional komité for medisinsk og helsefaglig forskningsetikk sør-øst Postboks 1130  0450 Oslo 0318  Norway | |
| 162/0235 | Regional komité for medisinsk og helsefaglig forskningsetikk sør-øst Postboks 1130  0450 Oslo 0318  Norway | |
| 162/0236 | Regional komité for medisinsk og helsefaglig forskningsetikk sør-øst Postboks 1130  0450 Oslo 0318  Norway | |
| 162/0237 | Regional komité for medisinsk og helsefaglig forskningsetikk sør-øst Postboks 1130  0450 Oslo 0318  Norway | |
| 162/0238 | Hospital Vall d'Hebrón  Dirección de investigación, planta 2 - Edifici Recerca  Passeig de la Vall d'Hebrón s/n 08035 Barcelona  Spain | |
| 162/0239 | Hospital Vall d'Hebrón  Dirección de investigación, planta 2 - Edifici Recerca  Passeig de la Vall d'Hebrón s/n 08035 Barcelona  Spain | |
| 162/0240 | Istanbul Medical Faculty Clinical Research Ethics Committee  Hulusi Behcet Library, floor 3 Capa-Fatih-Istanbul 34380 Istanbul  Turkey | |
| 162/0241 | Istanbul Medical Faculty Clinical Research Ethics Committee  Hulusi Behcet Library, floor 3 Capa-Fatih-Istanbul 34380 Istanbul  Turkey | |
| 162/0243 | Istanbul Medical Faculty Clinical Research Ethics Committee  Hulusi Behcet Library, floor 3 Capa-Fatih-Istanbul 34380 Istanbul  Turkey | |
| 162/0244 | Istanbul Medical Faculty Clinical Research Ethics Committee  Hulusi Behcet Library, floor 3 Capa-Fatih-Istanbul 34380 Istanbul  Turkey | |
| 162/0246 | Istanbul Medical Faculty Clinical Research Ethics Committee  Hulusi Behcet Library, floor 3 Capa-Fatih-Istanbul 34380 Istanbul  Turkey | |
| 162/0247 | Istanbul Medical Faculty Clinical Research Ethics Committee  Hulusi Behcet Library, floor 3 Capa-Fatih-Istanbul 34380 Istanbul  Turkey | |
| 162/0249 | Istanbul Medical Faculty Clinical Research Ethics Committee  Hulusi Behcet Library, floor 3 Capa-Fatih-Istanbul 34380 Istanbul  Turkey | |
| 162/0250 | Istanbul Medical Faculty Clinical Research Ethics Committee  Hulusi Behcet Library, floor 3 Capa-Fatih-Istanbul 34380 Istanbul  Turkey | |
| 162/0253 | Regionala etikprövningsnämnden i Uppsala Drottninggatan 4  753 09 Uppsala Sweden | |
| 162/0254 | Regionala etikprövningsnämnden i Uppsala Drottninggatan 4  753 09 Uppsala Sweden | |
| 162/0256 | Regionala etikprövningsnämnden i Uppsala Drottninggatan 4  753 09 Uppsala Sweden | |
| 162/0257 | Regionala etikprövningsnämnden i Uppsala Drottninggatan 4  753 09 Uppsala Sweden | |
| 162/0258 | Regionala etikprövningsnämnden i Uppsala Drottninggatan 4  753 09 Uppsala Sweden | |
| 162/0259 | Regionala etikprövningsnämnden i Uppsala Drottninggatan 4  753 09 Uppsala Sweden | |
| 162/0260 | Multicentrická Etická komisia Košického samosprávneho kraja  Námestie Maratónu mieru 1 Košice 042 66  Slovakia  Etická komisia ilinského samosprávneho kraja  Ul. Komenského 48  011 09 Ilina Slovakia | |
| 162/0261 | Multicentrická Etická komisia Košického samosprávneho kraja  Námestie Maratónu mieru 1 Košice 042 66  Slovakia  Etická komisia ilinského samosprávneho kraja  Ul. Komenského 48  011 09 Ilina Slovakia | |
| 162/0262 | Multicentrická Etická komisia Košického samosprávneho kraja  Námestie Maratónu mieru 1 Košice 042 66  Slovakia | |
| 162/0263 | Multicentrická Etická komisia Košického samosprávneho kraja  Námestie Maratónu mieru 1 Košice 042 66  Slovakia | |
| 162/0264 | Multicentrická Etická komisia Košického samosprávneho kraja  Námestie Maratónu mieru 1 Košice 042 66  Slovakia | |
| 162/0265 | Multicentrická Etická komisia Košického samosprávneho kraja  Námestie Maratónu mieru 1 Košice 042 66  Slovakia  Etická komisia Trenèianskeho samosprávneho kraja  K dolnej stanici 7282/20A 911 01 Trenèín  Slovakia | |
| 162/0267 | Multicentrická Etická komisia Košického samosprávneho kraja  Námestie Maratónu mieru 1 Košice 042 66  Slovakia  Etická komisia Nitrianskeho samosprávneho kraja  Štefánikova tr. 69  949 01 Nitra Slovakia | |
| 162/0268 | Multicentrická Etická komisia Košického samosprávneho kraja  Námestie Maratónu mieru 1 Košice 042 66  Slovakia  Etická komisia Bratislavského samosprávneho kraja Sabinovská 16  P.O. Box 106  820 05 Bratislava 25 Slovakia | |
| 162/0269 | Multicentrická Etická komisia Košického samosprávneho kraja  Námestie Maratónu mieru 1 Košice 042 66  Slovakia  Etická komisia Bratislavského samosprávneho kraja Sabinovská 16  P.O. Box 106  820 05 Bratislava 25 Slovakia | |
| 162/0270 | Multicentrická Etická komisia Košického samosprávneho kraja  Námestie Maratónu mieru 1 Košice 042 66  Slovakia  Etická komisia Nitrianskeho samosprávneho kraja  Štefánikova tr. 69  949 01 Nitra Slovakia | |
| 162/0271 | Multicentrická Etická komisia Košického samosprávneho kraja  Námestie Maratónu mieru 1 Košice 042 66  Slovakia  Etická komisia Prešovského samosprávneho kraja  Námestie mieru 2  080 01 Prešov Slovakia | |
| 162/0272 | METOPP  Beethovenlaan 332 A  5011 LN Tilburg Netherlands | |
| 162/0273 | METOPP  Beethovenlaan 332 A  5011 LN Tilburg Netherlands | |
| 162/0274 | METOPP  Beethovenlaan 332 A  5011 LN Tilburg Netherlands | |
| 162/0275 | METOPP  Beethovenlaan 332 A  5011 LN Tilburg Netherlands | |
| 162/0276 | Egészségügyi Tudományos Tanács Klinikai Farmakológiai Etikai Bizottsága  Arany János u. 6-8. 1051 Budapest Hungary | |
| 162/0277 | Egészségügyi Tudományos Tanács Klinikai Farmakológiai Etikai Bizottsága  Arany János u. 6-8. 1051 Budapest Hungary | |
| 162/0278 | Ethikkommission der Landesärztekammer Hessen  Im Vogelsgesang 3 60488 Frankfurt a. M Germany | |
| 162/0279 | Meir Hospital Tchernihovski St. 59  44281 Kfar Saba Israel | |
| 162/0281 | Comité de Ética en Investigación Clínica Dr. Carlos A. Barclay  Larrea 1381 3° A C1117ABK Buenos Aires Argentina  Comité de Bioética de MD Investigaciones Av. Balbin 3272. 5° C  C1430AAQ Buenos Aires Argentina | |
| 162/0282 | Comité de Ética de Protocolos de Investigación  Gascon 450  C1181ACH Buenos Aires Argentina | |
| 162/0283 | Comite de Etica de investigacion Hospital DIPRECA  Comite Etico Cientifico del Servicio de Salud Metropolitano Oriente  Avenue Salvador #364  7500922 Providencia Santiago Chile | |
| 162/0284 | BIOS- Comité de Etica en Investigacion de la Fundacion del Caribe para la Investigación Biomedica  Carrera 50 No. 80-216, piso 2, Oficina 203 Barranquilla  Colombia | |
| 162/0285 | Comité de Ética en Investigación Clínica Dr. Carlos A. Barclay  Larrea 1381 3° A C1117ABK Buenos Aires Argentina  Comité Docencia e Investigación Hospital Pilar 950 C1408INH Buenos Aires Argentina | |
| 162/0286 | IRB Services Hollandview Trail 372  Suite 300  L4G 0A5 Aurora Canada | |
| 162/0287 | Multicentrická Etická komisia Košického samosprávneho kraja  Námestie Maratónu mieru 1 Košice 042 66  Slovakia  Etická komisia ilinského samosprávneho kraja  Ul. Komenského 48  011 09 Ilina Slovakia | |
| 162/0288 | Comitato Etico della Provincia di Ferrara Via Aldo Moro 8  44124 Ferrara Italy | |
| 162/0289 | Edith Wolfson Medical Center Halochamim Street 62  58100 Holon Israel | |
| 162/0290 | Sredisnje Eticko Povjerenstvo Ksaverska cesta 4  10000 Zagreb Croatia | |
| 162/0291 | Komisja Bioetyczna przy Okregowej Izbie Lekarskiej  ul. Sniadeckich 33  80-204 Gdansk Poland | |
| 162/0292 | Komisja Bioetyczna przy Okregowej Izbie Lekarskiej  ul. Sniadeckich 33  80-204 Gdansk Poland | |
| 162/0293 | Komisja Bioetyczna przy Okregowej Izbie Lekarskiej  ul. Sniadeckich 33  80-204 Gdansk Poland | |
| 162/0295 | Komisja Bioetyczna przy Okregowej Izbie Lekarskiej  ul. Sniadeckich 33  80-204 Gdansk Poland | |
| 162/0297 | Komisja Bioetyczna przy Okregowej Izbie Lekarskiej  ul. Sniadeckich 33  80-204 Gdansk Poland | |
| 162/0301 | Ethikkommission der Landesärztekammer Hessen  Im Vogelsgesang 3 60488 Frankfurt a. M Germany | |
| 162/0302 | University Hospital Olomouc  I.P. Pavlova 6 775 20 Olomouc Czech Republic | |
| 162/0303 | University Hospital Olomouc  I.P. Pavlova 6  775 20 Olomouc Czech Republic | |
| 162/0304 | Komisja Bioetyczna przy Okregowej Izbie Lekarskiej  ul. Sniadeckich 33  80-204 Gdansk Poland | |
| 162/0305 | Comisia Nationala de Etica pentru Studiul Clinic al Medicamentului str. Av. Sanatescu nr. 48, sector 1  011478 Bucharest Romania | |
| 162/0306 | Comisia Nationala de Etica pentru Studiul Clinic al Medicamentului str. Av. Sanatescu nr. 48, sector 1  011478 Bucharest Romania | |
| 162/0309 | Comisia Nationala de Etica pentru Studiul Clinic al Medicamentului str. Av. Sanatescu nr. 48, sector 1  011478 Bucharest Romania | |
| 162/0310 | Komisja Bioetyczna przy Okregowej Izbie Lekarskiej  ul. Sniadeckich 33  80-204 Gdansk Poland | |
| 162/0311 | University Hospital Olomouc  I.P. Pavlova 6 775 20 Olomouc Czech Republic | |
| 162/0312 | University Hospital Olomouc  I.P. Pavlova 6  775 20 Olomouc Czech Republic | |
| 162/0313 | University Hospital Olomouc  I.P. Pavlova 6 775 20 Olomouc Czech Republic | |
| 162/0320 | Comisia Nationala de Etica pentru Studiul Clinic al Medicamentului str. Av. Sanatescu nr. 48, sector 1  011478 Bucharest Romania | |
| 162/0321 | Comisia Nationala de Etica pentru Studiul Clinic al Medicamentului str. Av. Sanatescu nr. 48, sector 1  011478 Bucharest Romania | |
| 162/0322 | Comisia Nationala de Etica pentru Studiul Clinic al Medicamentului str. Av. Sanatescu nr. 48, sector 1  011478 Bucharest Romania | |
| 162/0327 | Comite de Bioetica- CIMeL - Centro de Investigacion Medica Lanus  Tucuman 1314 B1824KAJ Buenos Aires Argentina | |
| 162/0330 | Comite de Etica en Investigacion Clinica Dr. Carlos A. Barclay  Larrea 1381 piso 3A C1117ABK Buenos Aires Argentina | |
| 162/0331 | Centro de Investigaciones Metabolicas - Comite de Etica en Investigacion  Viamonte 2278/80 C1056ABJ Buenos Aires Argentina | |
