# Supplementary material for: Patient and physician factors influence decision-making in hypercholesterolemia: a questionnaire-based survey
Source: Lipids Health Dis. 2015 May 19;14:45. doi: 10.1186/s12944-015-0037-y (PMC4457981; doi:10.1186/s12944-015-0037-y)
Supplement: Additional file 4: — Physician pragmatic use questionnaire. [file 12944_2015_37_MOESM4_ESM.doc]

Additional File 4 Physician pragmatic use questionnaire.
